# Honeycomb-like porous chitosan films prepared *via* phase transition of poly(*N*-isopropylacrylamide) during water evaporation under ambient conditions†

**DOI:** 10.1039/d0ra03845h

**Published:** 2020-05-26

**Authors:** H. Izawa, H. Kajimoto, M. Morimoto, H. Saimoto, S. Ifuku

**Affiliations:** Department of Chemistry and Biotechnology, Faculty of Engineering, Tottori University 4-101 Koyama-Minami Tottori 680-8550 Japan; Center for Research on Green Sustainable Chemistry, Tottori University Tottori 680-8550 Japan; Division of Instrumental Analysis, Research Center for Bioscience and Technology, Tottori University Tottori 680-8550 Japan

## Abstract

Honeycomb-like porous chitosan (CS) films are attractive tools for developing functional materials for filters, catalyses, adsorbents, biomaterials, *etc.* A simple method for fabricating honeycomb-like porous CS films without special reagents, facilities, and techniques would make them accessible. Here we introduce an easily available method for fabricating honeycomb-like CS films without a strong acid/base, toxic reagents, or special facilities/techniques. An aqueous solution containing CS and poly(*N*-isopropylacrylamide) (PNIPAm) was allowed to stand at 25 °C to evaporate water. After 3 days, CS–PNIPAm composite films with homogenously phase-separated PNIPAm particles were obtained. The PNIPAm particles were removed by immersion in methanol, and the resulting films dried under reduced pressure to become honeycomb-like porous CS films. The pore size could be varied in the range of 0.5–3.0 μm by altering the CS concentration and the molecular weight of CS where the pore size was reduced under conditions with stronger interaction between CS molecules. We reveal that the key to success with this system is the decrease of lower critical solution temperature (LCST) of PNIPAm with water evaporation. In addition, we confirmed the removed PNIPAm was recyclable in this system. Furthermore, we found this method was also applicable to alginate. The proposed facile method for fabricating honeycomb-like porous polymeric films could provide various functional porous materials.

## Abstract 2

A simple method for fabricating honeycomb-like porous chitosan films without special reagents, facilities, and techniques was achieved by using poly(*N*-isopropylacrylamide).

## Graphical abstract

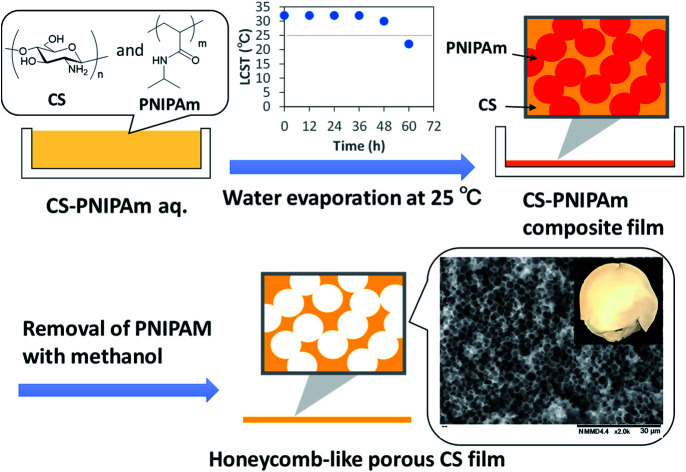

## Introduction

Macroporous polymer films with interconnected macropores (>50 nm)^1^ have attracted a great deal of attention, not only from separation science,^2–4^ but also catalysis, battery,^5,6^ and biomaterial fields,^7,8^ because they can be used as supports for catalysis, drugs, or living cells, and as frameworks^9^ in the fabrication of hierarchical porous materials.

Honeycomb structures inspired by bee honeycombs are a superior design used in various fields including architecture, and mechanical, acoustic, and chemical engineering, because they offer lighter material, high structural stability, large surface area, high permeability, and heat and sound-insulation.^10^ The marriage of the honeycomb structure to materials science has provided sophisticated macroporous materials. The breath figure method is a well-known approach to preparing monolayer honeycomb porous films, so-called honeycomb films, with hexagonally arranged pores ranging from sub μm to several tens of μm.^11–13^ The honeycomb films are applicable for surface enhanced Raman scattering (SERS),^14^ precise separation,^15^ optical^16^ and electronic devices,^17^ battery materials,^18,19^ biomaterials,^20,21^ and templates^22–24^ to prepare functional materials. On the other hand, porous films with a honeycomb-like ordered or disordered 3D interconnected network of monodisperse holes have been prepared *via* removal of colloid crystals^25,26^ or monodispersed particles and emulsions,^27,28^ respectively.

Chitosan (CS) is an amino polysaccharide produced by deacetylation of chitin.^29^ Although chitin is insoluble in both acid and alkaline aqueous solutions as well as in common organic solvents due to its strong hydrogen bonding, CS is soluble in acidic aqueous solution because of the protonation of the primary amino groups.^30^ Therefore, preparation of CS-based porous materials for adsorption of heavy metals, pollutant dyes, and proteins,^31–33^ as thermal insulators,^34^ and as biomaterials for wound healing,^35^ haemostasis,^36^ and tissue engineering^37^ can be achieved *via* freeze drying,^38^ supercritical CO_2_ drying,^34,39^ and the template-imprinting^32,40^ methods.

Recently, we accidently discovered the homogeneous phase separation of poly(*N*-isopropylacrylamide) (PNIPAm), a well-known thermoresponsive polymer showing a lower critical solution temperature (LCST) at *ca.* 32 °C in an aqueous solution,^41,42^ in a CS cast film prepared *via* water evaporation at room temperature. Although numerous CS–PNIPAm composites including PNIPAm-grafted CS derivatives were developed,^43–46^ the homogeneous phase separation of PNIPAm in a CS cast film was not reported. Furthermore, although honeycomb-like CS films with 0.1–1.0 μm pores prepared *via* the template-imprinting method with scarified polystyrene colloid crystals have been reported,^32^ the phenomenon we found provides a novel strategy for fabricating honeycomb-like CS films, *i.e.*, the homogenously phase-separated PNIPAm particles in the CS film are used as templates to make honeycomb-like pores.

Here, we investigate the preparation of honeycomb-like CS films *via* phase transition of PNIPAm during water evaporation at 25 °C and subsequent removal of PNIPAm particles to establish a novel methodology for fabricating honeycomb-like porous CS films. The mechanisms for the production of the honeycomb-like pores are clarified by analysis of LCST changes during water evaporation. We consider the effect of the CS/PNIPAm feed ratio and molecular weights of PNIPAm and CS on pore morphology and size. In addition, we apply this method to polyvinyl alcohol (PVA) and sodium alginate (AG).

## Experimental section

### Materials

Chitosans (CSs) were supplied by Koyo Chemical Co (Tottori, Japan) (*M*_n_ values estimated by GPC analysis with Pullulan standards were 64.1 × 10^3^ (*M*_w_/*M*_n_ = 2.03) and 162.4 × 10^3^ (*M*_w_/*M*_n_ = 1.98); degrees of deacetylation estimated by elemental analysis were 76.5% and 74.0%, respectively). Poly(*N*-isopropylacrylamide)s (PNIPAms) (*M*_n_: 40 000; *M*_n_: 85 000), amine terminated poly(*N*-isopropylacrylamide) (*M*_n_: 2500), and polyacrylamide (PAm) (*M*_n_: 40 000) were purchased from Sigma-Aldrich Japan (Tokyo). Sodium alginate (AG) (80–120 cps, 10 g L^−1^ at 25 °C), polyvinyl alcohol (PVA) (degree of polymerization: 1500) were purchased from Wako Pure Chemical Industries (Osaka, Japan). Other reagents were obtained in commercial grade and used without further purification.

### Instruments

SEM images were recorded using a TM303Plus (Hitachi, Japan) without coating. Nuclear magnetic resonance (NMR) spectra were recorded on a JNM-ECP500 (JEOL, Tokyo). UV-Vis spectra were recorded on a Multiskan GO (Thermo Fischer Scientific, Waltham, MA, USA).

### Preparation of the honeycomb-like porous films

CS and PNIPAm aqueous solutions were prepared as follows: CS (2.0 g) and acetic acid (0.5 g) were dissolved in 97.5 mL of water, and PNIPAm (2.0 g) was dissolved in 98 mL of water. Then, 5.0 g of the CS solution and 5.0 g of the PNIPAM solution were added to a Teflon Petri dish (*φ* = 50 mm). The CS–PNIAm solution was stirred, followed by degassing under reduced pressure. It was then kept at 25 °C for 72 h to yield a CS–PNIPAm composite film. The composite film was soaked in methanol (200 mL) for 24 h, and then dried under reduced pressure.

### GPC analysis of chitosans

*M*_n_ and *M*_w_/*M*_n_ values of CSs were measured by gel permutation chromatography (GPC) at 40 °C in acetate buffer solution eluent: Asahipak GS-220 HQ, Asahipak GS-320 HQ, Asahipak GS-520 HQ, and Asahipak GS-2G 7B (Shodex, Japan), a pump L-2130, and an RI-detector L-2490 (Hitachi, Japan). The flow rate was 0.5 mL min^−1^.

### Porosity of the honeycomb-like porous films

The weight-measured porous films were soaked in methanol and degassed under reduced pressure, then kept at 25 °C for 24 h. The weights of the methanol-adsorbed films were measured. The weight of adsorbed methanol was estimated from the weight difference between before and after methanol immersion. The porosities were calculated using eqn (1):1
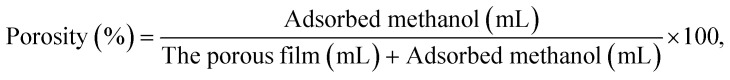
where the porous film (mL) and adsorbed methanol (mL) were calculated with the following equations: porous film weight (g)/1.425 (ref. 47) (CS density) and adsorbed methanol (g)/0.792 (methanol density), respectively. The theoretical porosities were calculated using eqn (2):2
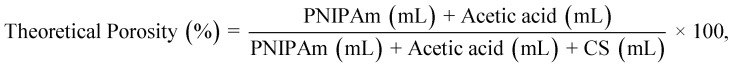
where the densities of CS,^47^ PNIPAm,^48^ and acetic acid used for the conversion of weight to volume were 1.425, 1.269, and 1.049, respectively.

## Results and discussion

### Preparation of the honeycomb-like porous CS film

**Fig. 1:**
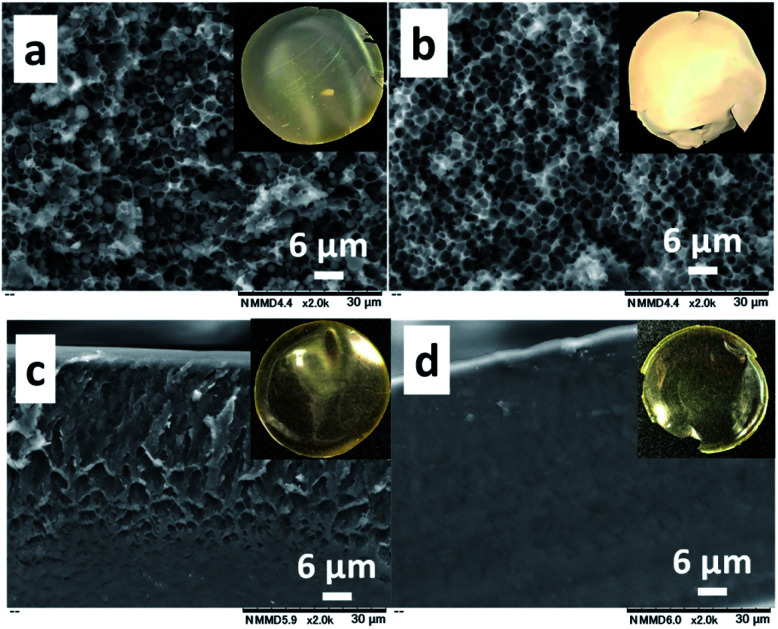
SEM images of the cross-sections of the obtained films with CS/PNIPAm (a and b) and CS/PAm (c and d) systems (1.0/1.0 in weight) before (a and c) and after (b and d) the methanol immersion. Multi-views are photos of the respective films. The *M*_n_ values of CS, PNIPAm, and PAm were 64.1 × 10^3^, 40.0 × 10^3^, and 40.0 × 10^3^, respectively.

The CS (*M*_n_: 64.1 × 10^3^)–PNIPAm (*M*_n_: 40.0 × 10^3^) aqueous solution (1.0/1.0 = CS/PNIPAm in weight) containing acetic acid was kept standing at 25 °C to produce a cast film. The cast film was soaked in methanol, which is a poor solvent for CS but has high affinity for CS. The resulting film was dried under reduced pressure. Fig. 1a and b show SEM and photo images of the obtained films before and after methanol immersion. The film before was hazy transparent, probably due to the phase separation of PNIPAm particles of *ca.* 2 μm inside, as shown in Fig. 1a. The particles were removed by methanol immersion, leaving behind the honeycomb-like film with *ca.* 2 μm pores. The ^1^H NMR spectrum of the materials removed by methanol showed PNIPAm and acetic acid (Fig. S4†), proving the observed particles in the film were PNIPAm. We carried out the experiment using a polyacrylamide (PAm) with a structure similar to PNIPAm, but without LCST (Fig. 1c and d). There were no particles/pores in the film before/after the methanol immersion, indicating that LCST-type phase separation of PNIPAm is related to the generation of honeycomb-like pores.

### Clarification of the mechanisms for the production of honeycomb-like porous CS films

**Fig. 2:**
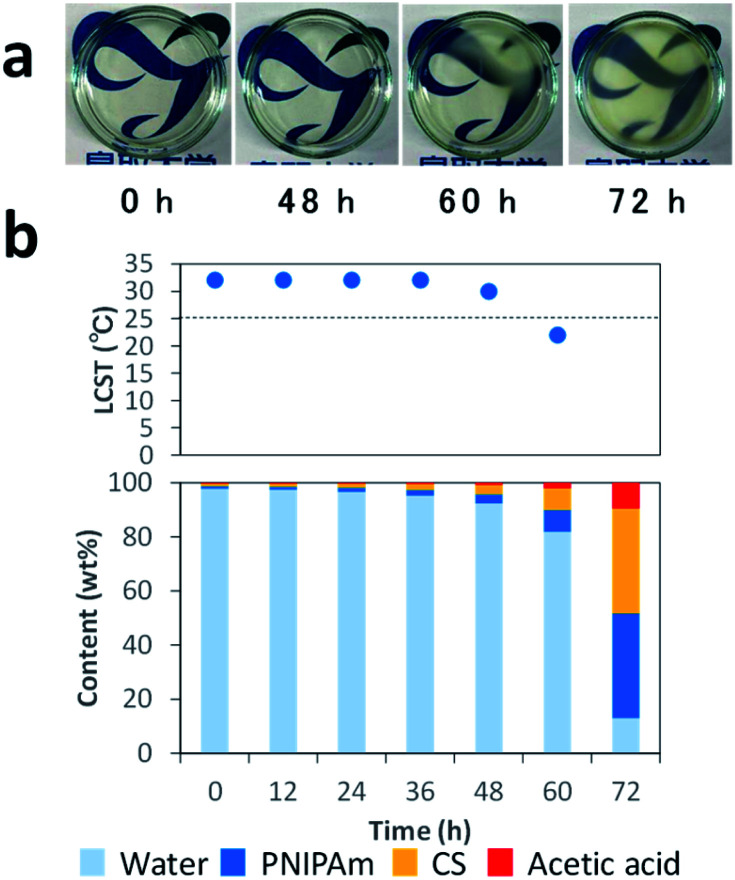
Photos of CS–PNIPAm solutions during water evaporation (a), and the solution contents and LCSTs of PNIPAm after 0, 12, 24, 36, 48, 60, and 72 h (b).

In order to clarify the mechanisms, we monitored the changes in appearance and weight during water evaporation at 25 °C. Fig. 2a presents photos of the solution during evaporation. The solution was transparent until 48 h, and then became partly hazy at 60 h due to the phase transition of PNIPAm. After 72 h, it became a cast film that we could pick up. Fig. 2b shows LCSTs of PNIPAm in the solution after 12, 24, 36, 48, 60, and 72 h, estimated from the clouding point measurement (Fig. S1†), and contents of the solution estimated from the weights of the solution at the respective times where it was assumed that the decreases of solution weights were caused only by water evaporation. The contents of CS (=PNIPAm) and acetic acid at 0, 12, 24, 36, 48, 60, or 72 h were 1.0, 1.2, 1.5, 2.1, 3.4, 8.1, or 38.8 wt% and 0.2, 0.3, 0.4, 0.5, 0.8, 2.0, or 9.5 wt%, respectively.

The LCSTs of PNIPAm in the solution at 0–36 h were 32 °C. After 48 and 60 h, the LCST decreased to 30 and 22 °C, respectively. The LCST after 60 h was below 25 °C, which was consistent with the observation of the phase transition. Indeed, the LCST of PNIPAm is decreased in high PNIPAm concentration,^49^ high salt concentration,^50^ and molecular crowding environments.^51^ Thus, the mechanisms of this system are as follows: CS, PNIPAm, and acetic acid concentrations gradually increase with water evaporation, which involves LCST decreases of PNIPAm. When LCST drops below 25 °C, the phase transition occurs in the highly viscous but homogeneous solution. This solution with homogenously phase-separated PNIPAm particles becomes the cast film with interconnected PNIPAm particles by the progress of water evaporation. Finally, the honeycomb-like pores are formed *via* the removal of PNIPAm with methanol. Thus, the key to this system is the decrease of LCST with water evaporation to generate the scarified PNIPAm particles.

### Effect of CS/PNIPAm feed ratio on honeycomb-like porous CS films

**Fig. 3:**
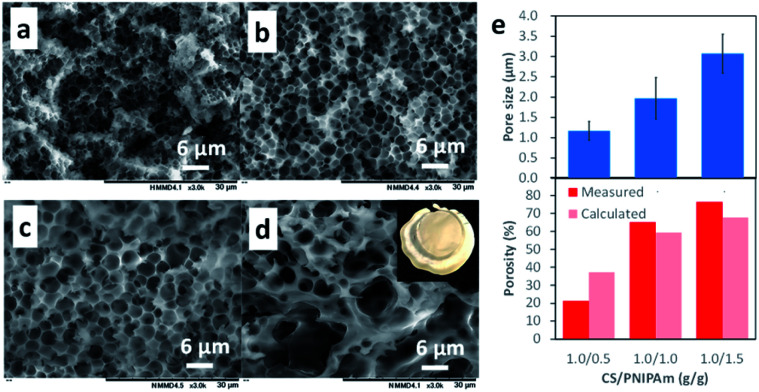
SEM images of the cross-sections of obtained films with different CS/PNIPAm feed ratios (1.0/0.5, 1.0/1.0, 1.0/1.5, and 1.0/2.0: (a), (b), (c), and (d), respectively), and their characterization (e). The *M*_n_ values of CS and PNIPAm were 64.1 × 10^3^ and 40.0 × 10^3^. Error bars indicate standard deviation.

The honeycomb-like porous CS films were prepared with various CS/PNIPAm feed ratios (1.0/0.5, 1.0/1.0, 1.0/1.5, and 1.0/2.0) while total polymer amounts (CS + PNIPAm) were held constant. Fig. 3 shows SEM images of the obtained films. In the case of the 1.0/2.0 feed ratio, an inhomogeneously shrunken film with inhomogeneous pores was observed. In the case of the 1.0/0.5, 1.0/1.0, and 1.0/1.5 feed ratios, the honeycomb-like pores were observed in the films. However, a substantial number of PNIPAm particles could also be discerned in the 1.0/0.5 feed ratio. Indeed, 6.3% of the unremoved PNIPAM was observed by ^1^H NMR spectra (Fig. S2†). In contrast, in the case of the 1.0/1.0 feed ratio, most PNIPAm was removed, and remaining PNIPAm in the porous film was estimated to be only 0.8%. This result indicates the PNIPAm particles isolated from the interconnected PNIPAm particles increased with the increase of CS, and could not be removed with methanol. The pore sizes and porosities in the 1.0/0.5, 1.0/1.0, or 1.0/1.5 feed ratios were 1.17 ± 0.51, 1.97 ± 0.51, or 3.07 ± 0.48 μm and 21.3%, 65.0%, or 76.4%, respectively (Fig. 3e). Pore size increased with the increase in PNIPAm feed ratio. In the case of the 1.0/0.5 feed ratio, porosity was lower than the theoretical value due to the presence of unremoved PNIPAm. However, the porosities for the 1.0/1.0 and 1.0/1.5 feed ratios were higher than their theoretical values. This was probably due to the removal of water remaining in the film by methanol immersion. Indeed, the porous film contained *ca.* 10 wt% water, as shown in Fig. 2b.

### Effect of molecular weight of CS and PNIPAm on pore size

**Fig. 4:**
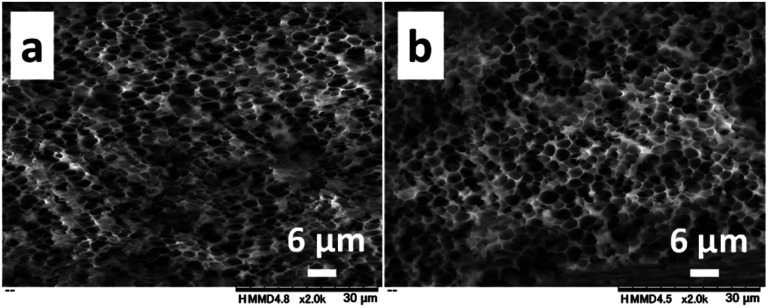
SEM images of the cross-sections of obtained films with the 1.0/1.0 feed ratio (CS/PNIPAm) using lower ((a) *M*_n_ = 2.5 × 10^3^) and higher ((b) *M*_n_ = 85.0 × 10^3^) molecular weights of PNIPAMs. The *M*_n_ of CS was 64.1 × 10^3^.

**Fig. 5:**
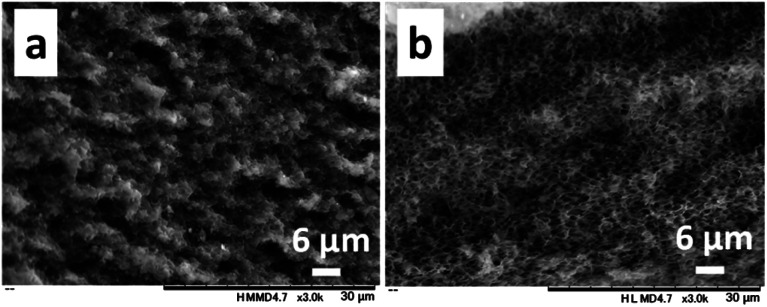
SEM images of the cross-sections of obtained films with the 1.0/1.0 (a), and 1.0/1.5 (b) feed ratios (CS/PNIPAm) using higher molecular weights of CS (*M*_n_ = 162.4 × 10^3^). The *M*_n_ of PNIPAm was 40.0 × 10^3^.

The effect of the molecular weight of PNIPAm and CS on pore sizes was investigated with the 1.0/1.0 feed ratio (CS/PNIPAm). When lower (*M*_n_: 2.5 × 10^3^) and higher (*M*_n_: 85.0 × 10^3^) molecular weights of PNIPAms were used (Fig. 4), pore sizes and porosities were almost the same as that of 40.0 × 10^3^ shown in Fig. 3b, indicating the molecular weight of PNIPAm was not an effective factor for controlling pore size. However, with higher molecular CS weights (*M*_n_: 162.4 × 10^3^), pore size decreased to 0.51 ± 0.14 μm, and the pores were not distinct, probably due to remaining PNIPAm (Fig. 5a). The amount of PNIPAm remaining in the porous film was estimated to be 10.3% (Fig. S3†). As a result, porosity was lower (44.3%) than the theoretical value (59.3%). In order to increase PNIPAm removal, the 1.0/1.5 feed ratio was employed (Fig. 5b). Although distinct pores were observed, they were larger (0.83 ± 0.17 μm) than those of the 1.0/1.0 feed ratio, as well as the system using the lower molecular weight CS shown in Fig. 3. Remaining PNIPAm and porosities were 1.6% and 76.0%, respectively. These results were also comparable to the results of the 1.0/1.5 feed ratio using the lower molecular weight CS shown in Fig. 3. They indicate we could prepare the honeycomb-like porous film with higher molecular weight CS, and that the molecular weight of CS is an effective factor for controlling pore size.

As described above, pore size decreased with an increase of CS feed ratio and molecular weight of CS; *i.e.*, the pore size was reduced under conditions with stronger interaction between CS molecules. This suggests the interaction between CS molecules is an important factor for controlling pore size.

### Recyclability of recovered PNIPAm

In this method, the PNIPAm particles were removed by washing with methanol, which is a non-destructive process for PNIPAm. Therefore, we investigated recyclability of the recovered PNIPAm. The methanol solution used for the removal of the PNIPAm particles was evaporated under reduced pressure. The ^1^H NMR spectrum of the resulting residue showed the presence of PNIPAm and a slight quantity of unevaporated acetic acid (Fig. S4†). The recovery of PNIPAm was 98.3%. We additionally confirmed that the recovered PNIPAm provided the same result as the original PNIPAm used as received (Fig. S5†).

### Application of this method to fabricate honeycomb-like porous PVA and AG films

**Fig. 6:**
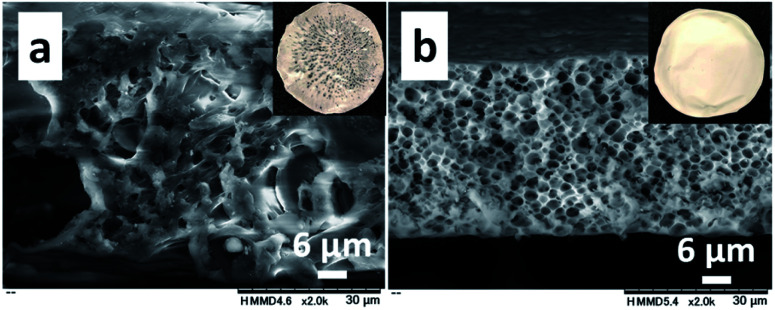
SEM images of the cross-sections of films obtained using the PVA/PNIPAm (a) and AG/PNIAm (b) systems (1.0/1.0). Multi-views are the photos of the respective films. The *M*_n_ of PNIPAm was 40.0 × 10^3^.

In order to investigate the versatility of this method, we applied it to PVA and AG (Fig. 6). In the case of PVA, we obtained an inhomogeneous film with irregular pores. In contrast, in the case of AG, a honeycomb-like AG film with 2.83 ± 0.87 μm pores was obtained. The CS and AG that enabled the honeycomb-like pores are both ionic polymers. These results suggest this system is applicable to water-soluble ionic polymers.

## Conclusions

We describe a novel methodology for fabricating honeycomb-like porous CS films. CS–PNIPAm aqueous solutions were kept standing at 25 °C. The LCST of PNIPAm was gradually decreased with water evaporation, and the phase transition of PNIPAm occurred when the LCST dropped below 25 °C. The CS–PNIPAm composite films with homogenously dispersed PNIPAm particles were obtained after 3 days. The honeycomb-like pores were formed *via* the removal of the PNIPAm particles by immersion in methanol. Pore size could be varied in the range of *ca.* 0.5–3.0 μm by changing the CS concentration and the molecular weight of CS. In addition, the removed PNIPAm was recyclable. Moreover, we revealed that this methodology was also applicable to AG. Although the template-imprinting method is the established approach for fabricating honeycomb-like porous polymeric films, this simple methodology constitutes a fabrication option that does not require a strong acid/base, toxic reagents, or special facilities/techniques; in addition, the PNIPAm can be recycled. The proposed method for fabricating honeycomb-like porous polymeric films could provide various functional porous materials.

## Conflicts of interest

There are no conflicts to declare.

## Supplementary Material

RA-010-D0RA03845H-s001
