# Supplementary material for: Honeycomb-like porous chitosan films prepared via phase transition of poly(N-isopropylacrylamide) during water evaporation under ambient conditions
Source: RSC Adv. 2020 May 26;10(34):19730–5. doi: 10.1039/d0ra03845h (PMC9054209; doi:10.1039/d0ra03845h)
Supplement: RA-010-D0RA03845H-s001 [file RA-010-D0RA03845H-s001.pdf]

Electronic Supplementary Material (ESI) for RSC Advances.
This journal is © The Royal Society of Chemistry 2020

Supplementary Information

Honeycomb-like porous chitosan films prepared via phase transition of poly(*N*-isopropylacrylamide) during water evaporation at ambient temperature

H. Izawa,[*,a,b] H. Kajimoto,[a] M. Morimoto,[c] H. Saimoto[a,b] and S. Ifuku[a,b]

[a] Department of Chemistry and Biotechnology, Faculty of Engineering, Tottori University, 4-101 Koyama-Minami, Tottori 680-8550, Japan.

[b] Center for Research on Green Sustainable Chemistry, Tottori University, Tottori 680-8550, Japan.

[c] Division of Instrumental Analysis, Research Center for Bioscience and Technology, Tottori University, Tottori 680-8550, Japan.

1. Cloud-point measurement with UV-Vis spectra

The cloud points of the solutions after 0, 12, 24, 36, 48, and 60 h were investigated (**Fig. S1**). Cloud points of the solutions were 32, 32, 32, 32, 30, and 22 ℃, respectively.

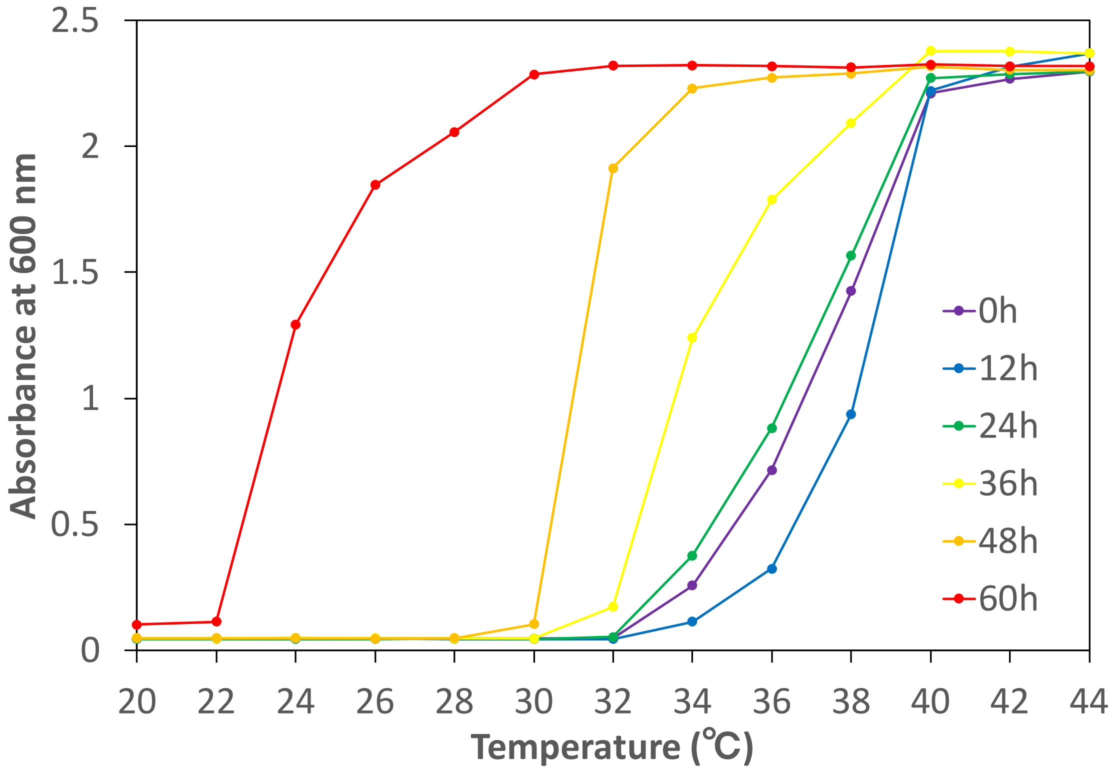

Fig. S1. Cloud-point measurements for CS-PNIPAm solutions after 0, 12, 24, 36, 48, and 60 h.

2. ^{1}H NMR analysis of the obtained films

The CS/PNIPAm ratios of the obtained porous films were estimated from the ^{1}H NMR analysis where the integration ratios of signals **g**, **e,** and **a** were used for the calculation. The CS/PNIPAm ratios were calculated using the following equation:

CS/PNIPAm (g/g) = GlcNAc unit (g) + GlcN unit (g) / PNIPAm (g) = $\mathbf{g} \times 203.3 + \mathbf{e} \times 161.1 / (a/6) \times 113.1$.

The amounts of remaining PNIPAms were estimated from the CS/PNIPAm ratio in the obtained films. These amounts in the 1.0/0.5, 1.0/1.0, and 1.0/1.5 feed ratios were 6.3%, 0.8%,

and 1.0%, respectively (**Fig. S2**). In the case of the 1.0/1.0 and 1.0/1.5 feed ratios using the higher molecular weight CS, the amounts of remaining PNIPAms were 10.3% and 1.6%, respectively (**Fig. S3**). Although most acetic acids were removed from the obtained films after the immersion into methanol as shown in **Fig. S2** and **S3**, we suppose slight quantities of acetic acids were included in the obtained films. However, their quantification with ^{1}H NMR analysis was difficult because the spectrum was overlapped by the signals attributed to PNIPAm (**b**) and CS (**h**).

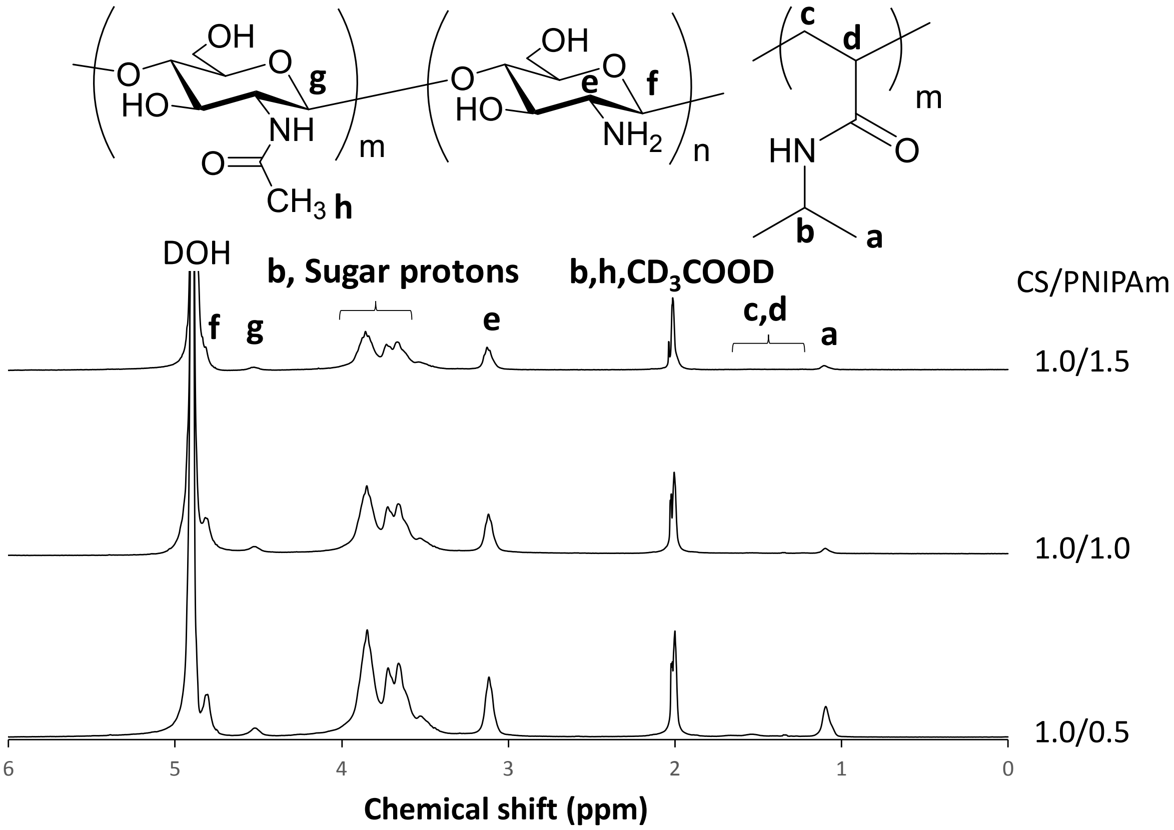

Fig. S2. ^{1}H NMR spectra of the obtained porous films with the 1.0/0.5, 1.0/1.0, and 1.0/1.5 feed ratios (CS/PNIPAm) in 10% CD_3COOD-D_2O. The M_n values of CS and PNIPAm were 64.1 x 10^3 and 40.0 x 10^3, respectively.

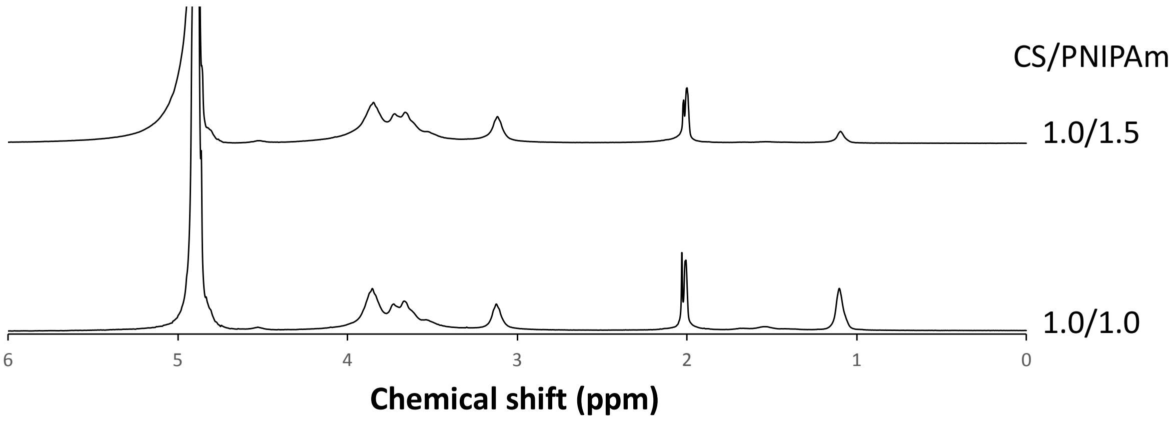

Fig. S3. ^{1}H NMR spectra of obtained porous films with the 1.0/1.0 and 1.0/1.5 feed ratios (CS/PNIPAm) using the higher molecular weight CS (M_n = 162.4 x 10^3) in 10% CD_3COOD-D_2O.

3. Recyclability of the recovered PNIPAm

The methanol solution containing the materials removed from the film was evaporated, and the residue was dried under reduced pressure. **Figure S4** shows the ^{1}H NMR spectrum of these removed materials. In the spectrum, PNIPAm and a slight amount of acetic acid were observed.

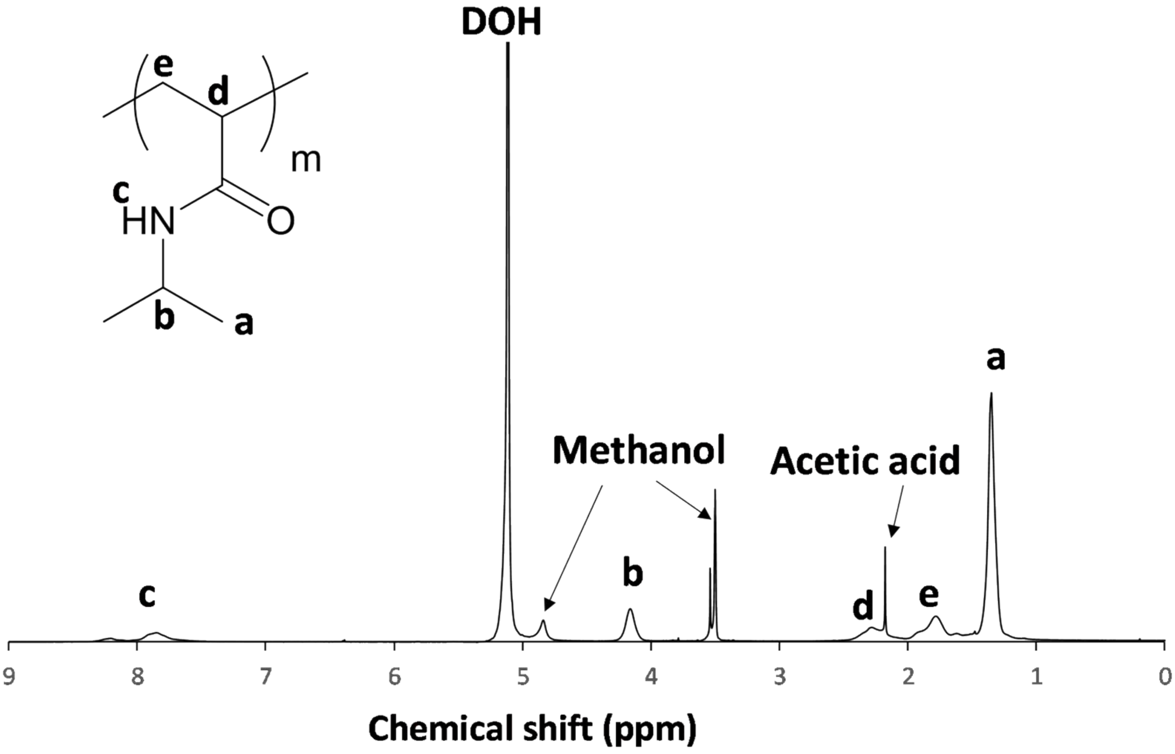

Fig. S4. ^{1}H NMR spectrum of the materials removed by methanol in the 1.0/1.0 system in CD_3OD. The M_n values of CS and PNIPAm were 64.1 x 10 and 40.0 x 10^3, respectively.

A honeycomb-porous film was prepared with the recovered PNIPAm (**Fig. S5**). The pores observed in the film were the same as those from the original PNIPAm.

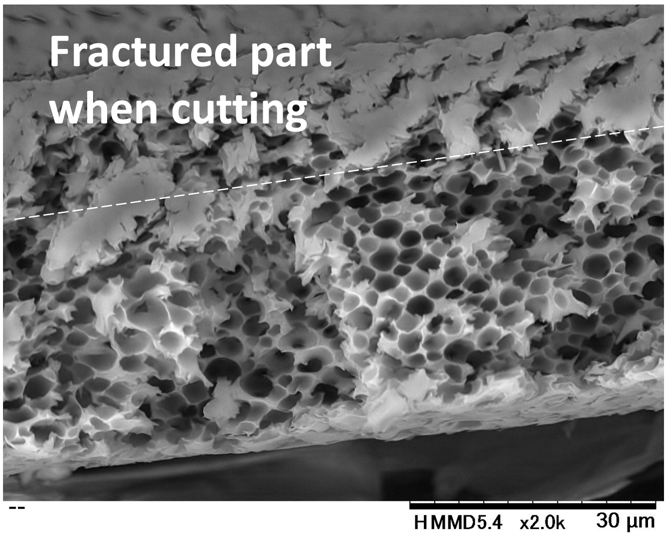

Fig. S5. The SEM image of the honeycomb-like porous film obtained with the 1.0/1.0 system using the recovered PNIPAm.
